# Prognostic value of pretreatment radiological MRI variables and dynamic contrast-enhanced MRI on radiotherapy treatment outcome in laryngeal and hypopharyngeal tumors

**DOI:** 10.1016/j.ctro.2024.100857

**Published:** 2024-09-12

**Authors:** Hilde J.G. Smits, Saskia J. Vink, Mischa de Ridder, Marielle E.P. Philippens, Jan W. Dankbaar

**Affiliations:** a Department of Radiotherapy, University Medical Center Utrecht, Utrecht, The Netherlands; b Department of Radiology, University Medical Center Utrecht, Utrecht, The Netherlands

**Keywords:** Dynamic Contrast-Enhanced MRI, Head and neck cancer, Prognostic study, Perfusion, Tumor volume

## Abstract

**Background:**

This study aimed to determine the prognostic value of radiological magnetic resonance imaging (MRI) variables and dynamic contrast enhanced (DCE)-MRI for local control (LC), disease control (DC), and overall survival (OS) in laryngeal and hypopharyngeal cancer patients after radiotherapy.

**Methods:**

320 patients treated with radiotherapy were retrospectively included. Pretreatment MRIs were evaluated for the following anatomical tumor characteristics: cartilage invasion, extralaryngeal spread, and involvement of the anterior commissure, pre-epiglottic space, and paralaryngeal space.

Pretreatment DCE-MRI was available in 89 patients. The median and 95th percentile of the 60-second area under the contrast-distribution-curve (AUC60_median_ and AUC60_p95_) were determined in the tumor volume.

**Results:**

Univariable log-rank test determined that extralaryngeal spread, tumor volume and T-stage were prognostic for worse LC, DC, and OS. A low AUC60_p95_ (<31.7 mmol·s/L) and thyroid cartilage invasion were prognostic for worse OS.

In multivariable analysis, a Cox proportional hazard model showed that a AUC60_p95_ ≥ 31.7 mmol·s/L was prognostic for better OS (HR=0.25, *P*<.001). Tumor volume was prognostic for DC (HR=3.42, *P*<.001) and OS (HR=3.27, *P*<.001). No anatomical MRI variables were significantly prognostic for LC, DC, or OS in multivariable analysis when corrected for confounders.

**Conclusion:**

Low pretreatment AUC60_p95_ is prognostic for a worse OS, suggesting that poor tumor perfusion leads to worse survival. Large tumor volume is also prognostic for worse DC and OS. Anatomical MRI parameters are not prognostic for any of the evaluated treatment outcomes when corrected for confounders like age, T-stage, N-stage, and tumor volume.

## Highlights

• The prognostic value of pre-radiotherapy DCE-MRI was analyzed in head and neck cancer.
• The area under the contrast concentration curve of 60 s (AUC60) was calculated.
• Low AUC60_p95_ is independently prognostic for a worse 5-year overall survival.
• Tumor volume is an independent prognostic variable when corrected for T-stage.

## Introduction

Radiotherapy is an often used organ-preserving treatment for laryngeal and hypopharyngeal tumors. However, not all patients respond to radiotherapy, so it is important to identify patients who are likely to benefit from concomitant therapy or surgical intervention. Pretreatment imaging can be used to identify quantitative prognostic factors in a minimally invasive manner.

In a recent systematic literature review, we identified prognostic imaging variables of recurrent laryngeal and hypopharyngeal carcinoma after (chemo)radiotherapy [1]. We found strong evidence for tumor volume as a prognostic factor, as well as anterior and posterior commissure involvement. Moderate and limited evidence was found for pre-epiglottic and paralaryngeal space involvement, extralaryngeal tumor extension and cartilage invasion.

Most studies included in this review were based on CT variables, as literature on the prognostic value of MR characteristics was limited for laryngeal and hypopharyngeal cancer. However, most of the anatomical subsites identified as prognostic factors on CT can be better assessed on MRI due to its superior soft tissue contrast [2]. This enables a better detection of subtle abnormalities in for example the pre-epiglottic and paralaryngeal space [2]. In this study, we assessed the prognostic value of radiological tumor characteristics as examined on MR.

Additionally, we examined the role of tumor perfusion on treatment outcome, which is thought be related to tumor hypoxia. Tumor hypoxia is associated with radiation resistance, tumor aggressiveness, and metastasis [3]. Poorly perfused tumors are thus more likely to lead to worse treatment outcomes.

Dynamic contrast-enhanced (DCE) MRI is a technique that depicts blood perfusion throughout a tumor. In DCE-MRI, a series of images is acquired after intravenous injection of a contrast agent [4]. The change in contrast agent over time can be used to estimate properties of the tumor vasculature. Pretreatment DCE-MRI might therefore be used to identify tumors that are more radio-resistant and have worse prognosis.

The aim of this study is to determine the prognostic value of pretreatment radiological MRI variables, including DCE-MRI, for radiotherapy treatment response in laryngeal and hypopharyngeal cancer patients.

## Methods

### Patient population

We retrospectively identified patients with laryngeal or hypopharyngeal carcinoma who were treated with radiotherapy between January 2009 and March 2020 in the University Medical Center Utrecht, the Netherlands. Patients who had previously been irradiated for head and neck carcinoma, or received post-operative or palliative radiotherapy were excluded. Other exclusion criteria were: distant metastases or other primary tumors at time of diagnosis, non-squamous cell carcinoma, carcinoma in situ, uncompleted radiotherapy treatment, a follow-up period < 2 years, or no available pretreatment MRI scan taken within 31 days of the start of treatment. The medical ethics committee waived the need for informed consent.

Clinical data was extracted from electronic patient files. The following factors were included: sex, age at the start of radiotherapy, tumor location (larynx, hypopharynx), and TNM-stage. Additionally, we collected the gross tumor volume (GTV) delineation used for radiotherapy treatment to determine the tumor volume. These GTVs were contoured on the planning CT by radiation oncologists, who also used rigidly registered MRI scans, PET-scans, and endoscopic reports to aid their delineation. Follow-up data was collected up to five years after treatment start.

### MR protocol

All patients underwent a preoperative MRI scan on either a 1.5T Intera (2009–2012) or a 3T Ingenia (2012–2020) MR system (Philips Healthcare, Best, The Netherlands), using two flexible surface coils and a posterior coil in the couch on 3T. Details regarding MR image acquisition can be found in Supplementary Material 1.

A five-point head-and-shoulder mask was used to immobilize patients. On 1.5T, T1-weighted scans before and after contrast administration and T2-weighted scans without fat suppression were acquired using a multi slice fast spin echo sequence. On 3T, T1-weighted scans without fat suppression, post-contrast agent T1-weighted multiple Dixon water images, and T2-weighted multiple Dixon water images were acquired with a multi slice fast spin echo sequence.

From December 2015 onwards, DCE-MRIs were also routinely acquired on 3T MRIs. Each DCE-MRI sequence consisted of a series of images that were acquired with a spoiled gradient echo and SPIR fat suppression. In most cases the temporal resolution was 4.95 s, but this ranged from 4.50 to 5.01 s.

The contrast agent gadobutrol (Gd-BT-DO3A, Gadovist; Schering AG, Berlin, Germany) was injected intravenously with a flow rate of 1 ml/s at a dosage of 0.1 mmol/kg of body weight of the patient, followed by a saline flush. Prior to the DCE-MRI scans, two scans were made with flip angles of 6° and 16° to estimate the pre-contrast native T1 values. These were used to convert the MRI signal intensity values to concentration of the contrast agent.

### Treatment and follow-up

All patients were treated with conventional radiotherapy using a two arc volumetric arc therapy technique with 6MV photons. For patients with T1 and limited T2 tumors without nodal disease, the prescribed total dose was 60 Gy in 25 fractions of 2.4 Gy. Patients with a T3 or T4 tumor and/or nodal disease received a total dose of 70 Gy in 35 fractions of 2 Gy. Patients received five or six fractions per week for a duration of five to seven weeks.

In addition to radiotherapy, some patients with higher staged tumors received concurrent chemotherapy (three cycles of intravenous cisplatin at a dose of 100 mg/m^2^) or targeted therapy (loading dose of cetuximab of 400 mg/m^2^ in the week preceding radiotherapy followed by a weekly dose of 250 mg/m^2^ for the duration of the treatment).

Follow-up consisted of regular visits with either a head and neck surgeon or a radiation oncologist for at least five years. All patients underwent response evaluation imaging (either MRI or CT-scan) at three months post-treatment. Follow-up visits occurred every two months in the first year, every three months in the second year, every four months in the third year and every six months in the last two years. At each visit, a physical examination took place with flexible laryngoscopy. Suspected recurrences were pathologically confirmed if the patient’s condition allowed.

### Anatomical tumor characteristics

The pretreatment MR images were evaluated by an experienced head-and-neck radiologist (J.W.D.), who determined the presence of the following tumor characteristics: anterior commissure involvement, cricoid cartilage invasion, posterior commissure involvement, pre-epiglottic space involvement, paralaryngeal space involvement, and extralaryngeal spread. Additionally, thyroid cartilage invasion was determined based on three categories [5]: tumor growth clear of cartilage, adjacent to cartilage, or tumor eroded or invaded the cartilage.

### DCE-MRI analysis

The workflow of DCE-MRI analysis used in this paper was based on the work of Heethuis et al. (2016) [6] and adapted for laryngeal and hypopharyngeal tumors. A detailed description of the workflow can be found in Supplementary Material 2.

To correct for breathing and swallowing motions with the DCE-MRI series, all scans were non-rigidly registered to a scan after contrast-enhancement using the Elastix toolbox [7], [8].

The signal intensity of the scans were converted to contrast agent concentration. The pre-contrast T1 relaxation times were calculated with in-house developed software. The concentrations were corrected with the gadobutrol relaxation rate constant of 4.5 L mmol^−1^ s^−1^ at a 3T field strength [9].

The DCE data was analyzed using the area under the contrast distribution curve for 60 s (AUC60). To determine the AUC60, we first selected the arterial input function (AIF) from the DCE-MRI of each patient. The AIF is a curve depicting the influx of contrast agent over time and was selected from the common or exterior carotid artery on the same side as the tumor. From the AIF, the peak of contrast influx was determined. One time point prior to the peak (circa five seconds) was chosen as the starting point of the AUC60 measure. We calculated the AUC60 for each voxel within the tumor area using Matlab R2019a.

Laryngeal and hypopharyngeal tumors are likely to border air cavities or cartilage. Since it is impossible to calculate the AUC60 in these areas, it is likely that the AUC60 measurements in voxels around the edges of the tumor contain artifacts due to residual motion in the DCE scans. To negate these errors, all voxels that contained concentration measurements smaller than the second percentile or larger than the 98th percentile at any time point were removed from analysis, Supplementary Material 2. Additionally, if the resulting AUC60 was negative, the voxel was removed from analysis. From the remaining voxels in the tumor area, the median and 95th percentile AUC60 value (AUC60_median_ and AUC60_p95_, respectively) were calculated and used in analysis.

### Statistical analysis

Statistical analysis was performed in R version 4.2.2 [10]. The GTV volume, and the median and p95 AUC60 were dichotomized into a high and low group. The surv_cutpoint function from the survminer R package was used to determine the best cutoff value [11].

In the univariable analysis, Kaplan-Meier curves were created to estimate the local control (LC), disease control (DC), and overall survival (OS). Events for these outcome measures were defined as local recurrence, any recurrence (local, regional or distant metastases) and death of any cause, respectively. The log-rank test was used to determine statistical significant variables. A Bonferroni correction was applied to reduce the risk of type I errors. *P*≤.05 after Bonferroni correction (0.05 divided by the number of risk factors included in univariable analysis) was considered a significant result.

Two models were created for the multivariable analysis. The first model was based on the anatomical MRI characteristics and clinical variables of the entire cohort. The second model was based on the subset of patients with a DCE scan and did not include anatomical MRI characteristics. Only the DCE variables and the clinical variables were included in this model. The Cox proportional hazards model was used with backward variable elimination to identify independent prognostic factors. *P*≤.05 after Bonferroni correction was considered a significant result. Furthermore, we used the general variance inflation factor (GVIF) to evaluate the presence of multicollinearity among all covariates for each variable.

## Results

In total, 320 patients were included in the final analysis. Patient and tumor characteristics are shown in Table 1. The median follow-up time of the surviving patients was 60 months (range: 27–60 months). In this time 96 patients (30 %) developed a tumor recurrence: 61 patients (19 %) had a local recurrence, 35 (11 %) a regional recurrence, and 40 (13 %) a distant metastasis. 137 patients (43 %) died. The 5-year LC, DC, and OS rates for the whole cohort were 81 %, 70 %, and 57 %, respectively.

**Table 1:** Patient and tumor characteristics.

**Patient and tumor characteristic**		**Complete dataset (n = 320)**		**DCE-MRI subset (n = 89)**	
		n	(%)	n	(%)
Sex	Male	243	(76)	65	(73)
	Female	77	(24)	24	(27)
Age [years]	Median	66		67	
	Range	40–94		43–91	
Tumor location	Larynx	246	(77)	69	(78)
	Hypopharynx	74	(23)	20	(22)
GTV volume [cm^3^]	Median	6.6		5.0	
	Range	0.3–154.7		0.3–38.1	
Tumor stage	T1	44	(14)	11	(12)
	T2	127	(40)	41	(46)
	T3	117	(37)	29	(33)
	T4a/b	27/5	(10)	8/0	(9)
Nodal stage	N0	193	(60)	50	(56)
	N1	42	(13)	14	(16)
	N2a/b/c	2/40/35	(24)	0/8/12	(22)
	N3	8	(3)	5	(6)
Days between MRI and start treatment	Median	14		18	
	Range	0–31		3–31	
Treatment	RT only	269	(84)	78	(88)
	RT+chemo	39	(12)	10	(11)
	RT+cetuximab	12	(4)	1	(1)
Thyroid cartilage invasion scale	Clear	153	(48)	47	(53)
	Adjacent	135	(42)	34	(38)
	Invasion	32	(10)	8	(9)
Cricoid cartilage invasion	No	318	(99)	89	(1 0 0)
	Yes	2	(1)	−	(0)
Anterior commissure involvement	No	233	(73)	64	(72)
	Yes	87	(27)	25	(28)
Posterior commissure involvement	No	318	(99)	89	(1 0 0)
	Yes	2	(1)	−	(0)
Pre-epiglottic space involvement	No	288	(90)	82	(92)
	Yes	32	(10)	7	(8)
Paralaryngeal space involvement	No	152	(48)	45	(51)
	Yes	168	(53)	44	(49)
Extralaryngeal spread	No	289	(90)	80	(90)
	Yes	31	(10)	9	(10)
AUC60_median_ [mmol·s/L]	Median	−		17.4	
	Range	−		5.8–34.4	
AUC60_p95_ [mmol·s/L]	Median	−		39.7	
	Range	−		17.5–66.4	
**Follow-up**					
Local Recurrence		61	(19)	15	(17)
Regional Recurrence		35	(11)	7	(8)
Distant Metastasis		40	(13)	11	(12)
Any recurrence		96	(30)	26	(29)
Death		137	(43)	34	(38)

Both cricoid cartilage invasion and posterior commissure involvement were only found in two patients, so these variables were excluded from further analysis. This left 14 risk factors in the univariable analysis. *P*≤.004 was considered a significant result.

**Fig. 1:**
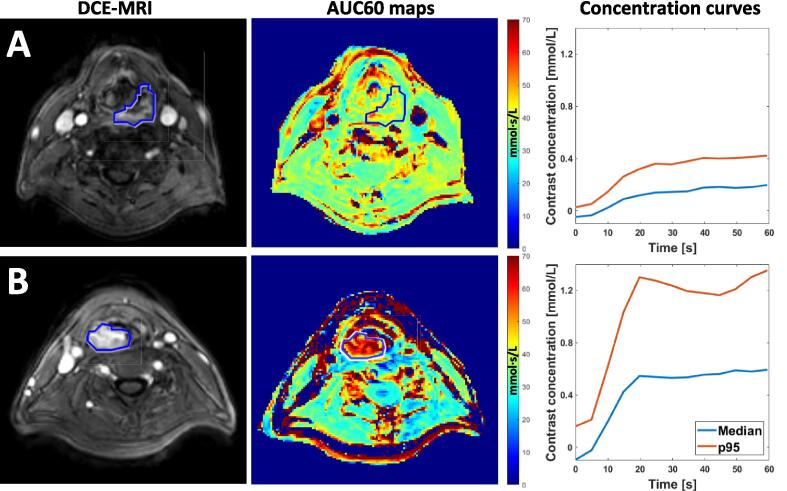
Examples of two patients with low (A) and high (B) tumor perfusion. The AUC60 maps show the area under the contrast concentration curve for the first 60 s after contrast injection for each voxel. The concentration curves show the median and 95th percentile gadolinium concentration in the tumor over time. Patient A shows limited tumor perfusion with an AUC60_median_ of 7.1 mmol·s/L, while patient B shows a high perfusion with an AUC60_median_ of 26.6 mmol·s/L.

Pre-treatment DCE-MRI was available in 97 patients. A further 7 patients were excluded due to DCE acquisition shorter than 60 s (4), no available clinical GTV (2), and incomplete imaging for T1 mapping (1). After removing voxels with abnormally high or low contrast measurements, there was one tumor in which only 3% of voxels remained. This patient was also excluded, leaving 89 patients that were included in the DCE-MRI analysis (Table 1, Fig. 1).

The clinical GTV delineation could not be accessed in four patients, so analysis of tumor volume as a risk factor was based on 316 patients. The cut-offs used to dichotomize the GTV volume, AUC60_median_ and AUC60_p95_ were 14.8 cm^3^, 21.7 mmol·s/L, and 31.7 mmol·s/L, respectively.

When checking for multicollinearity, paralaryngeal space involvement had a high GVIF and was found to be strongly correlated to thyroid cartilage invasion, Supplementary Material 3. To avoid unwanted effects, paralaryngeal space involvement was excluded from multivariable analysis.

**Fig. 2:**
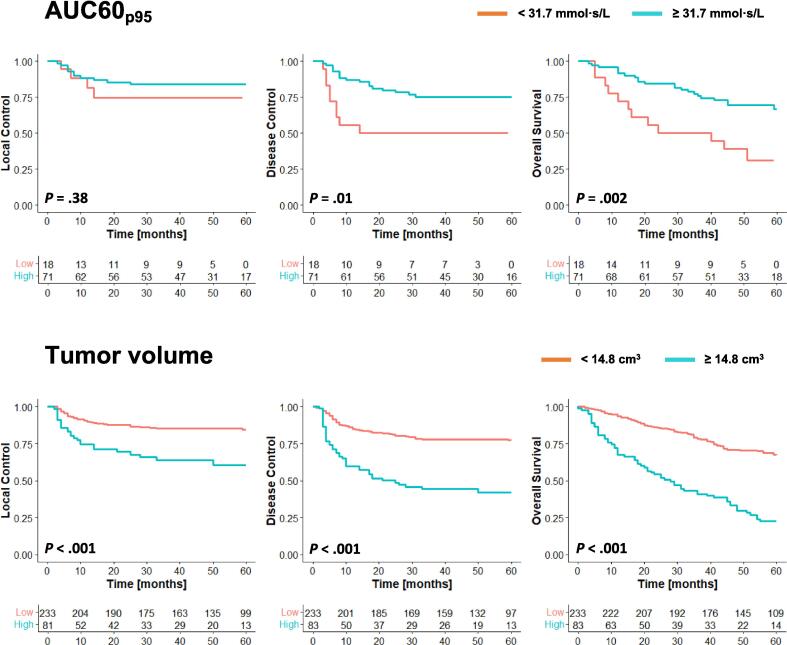
Kaplan-Meier survival curves of local control, disease control and overall survival stratified according to the 95th percentile of the area under the concentration curve of the first 60 s (AUC60_p95_) and tumor volume. The tables below show the number of at risk.

Kaplan-Meier plots of AUC60_p95_ and tumor volume for all outcomes can be found in Fig. 2.

### Local control

In the univariable analysis, the following factors were prognostic for a worse LC: tumor volume ≥ 14.8 cm^3^, tumor stage T4a compared to tumor stage T1, and extralaryngeal spread (Table 2). In the multivariable analysis, no significant variables were found in both the anatomical and the DCE model.

**Table 2:** Univariable analyses of prognostic factors for local control, disease control, and overall survival.

**Patient and tumor characteristics**		Patients		**Local Control**					**Disease Control**					**Overall Survival**			
				Events	HR	95 % CI	*P*-value		Events	HR	95 % CI	*P*-value		Events	HR	95 % CI	*P*-value
Sex	Male	243		52	−				75	−				108	−		
	Female	77		9	0.52	0.26–1.05	.07		21	0.84	0.52–1.36	.48		29	0.79	0.52–1.19	.26
Age [years]	<65	149		27	−				39	−				44	−		
	≥65	171		34	1.18	0.71–1.95	.53		57	1.40	0.93–2.11	.10		93	2.24	1.56–3.21	**<.001**
Tumor location	Larynx	246		45	−				66	−				98	−		
	Hypopharynx	74		16	1.30	0.73–2.30	.37		30	1.76	1.15–2.72	.01		39	1.55	1.07–2.25	.02
GTV volume [cm^3^]	<14.8	233		34	−				51	−				74	−		
	≥14.8	83		26	2.94	1.76–4.91	**<.001**		44	3.42	2.28–5.13	**<.001**		63	3.76	2.68–5.27	**<.001**
Tumor stage	T1	44		4	−				8	−				10	−		
	T2	127		19	1.74	0.59–5.11	.31		33	1.53	0.71–3.32	.28		45	1.63	0.82–3.23	.16
	T3	117		25	2.65	0.92–7.62	.07		36	1.89	0.88–4.07	.10		57	2.54	1.30–4.98	0.01
	T4a	27		12	7.19	2.32–22.33	**.001**		16	4.88	2.09–11.42	**<.001**		20	5.34	2.50–11.42	**<.001**
	T4b	5		1	5.43	0.60–48.81	.13		3	8.02	2.12–30.41	**.002**		5	16.27	5.52–47.90	**<.001**
Nodal stage	N0	193		31	−				42	−				59	−		
	N+	127		30	1.78	1.08–2.94	.03		54	2.43	1.62–3.63	**<.001**		78	2.71	1.93–3.81	**<.001**
Treatment	RT only	269		49	−				72	−				103	−		
	RT+chemo	39		8	1.24	0.59–2.61	.58		16	1.67	0.97–2.87	.06		23	1.80	1.15–2.83	.01
	RT+cetuximab	12		4	3.02	1.09–8.37	.03		8	3.93	1.89–8.18	**<.001**		11	4.26	2.28–7.95	**<.001**
Thyroid cartilage invasion scale	Clear	153		24	−				37	−				53	−		
	Adjacent	135		28	1.42	0.83–2.46	.21		44	1.47	0.95–2.28	.08		63	1.49	1.15–2.83	.03
	Invasion	32		9	2.11	0.98–4.55	.06		15	2.35	1.29–4.28	.01		21	2.50	1.51–4.16	**<.001**
Anterior commissure involvement	No	233		42	−				71	−				104	−		
	Yes	87		19	1.19	0.69–2.04	.53		25	0.91	0.58–1.44	.69		33	0.80	0.54–1.18	.26
Pre-epiglottic space involvement	No	288		51	−				83	−				119	−		
	Yes	32		10	2.10	1.07–4.14	.03		13	1.71	0.95–3.08	.07		18	1.68	1.02–2.76	.04
Paralaryngeal space involvement	No	152		24	−				36	−				53	−		
	Yes	168		37	1.53	0.92–2.57	.10		60	1.68	1.11–2.55	.01		84	1.64	1.16–2.31	.01
Extralaryngeal spread	No	289		50	−				79	−				114	−		
	Yes	31		11	2.84	1.48–5.47	**.002**		17	2.89	1.71–4.88	**<.001**		23	2.96	1.89–4.64	**<.001**
AUC60_median_ [mmol·s/L]	<21.7	68		12	−				23	−				31	−		
	≥21.7	21		3	0.72	0.20–2.56	.62		3	0.35	0.11–1.18	.09		3	0.24	0.07–0.80	.02
AUC60_p95_ [mmol·s/L]	<31.7	18		4	−				9	−				12	−		
	≥31.7	71		11	0.60	0.19–1.89	.38		17	0.35	0.15–0.78	.01		22	0.33	0.16–0.67	**.002**

Significant *P*-values are bold. Abbreviations: HR, hazard ratio; CI, confidence interval; RT, radiotherapy.

### Disease control

In the univariable analysis, the following factors were prognostic for a worse DC: tumor volume ≥ 14.8 cm^3^, tumor stage T4a and T4b compared to tumor stage T1, nodal disease, concurrent cetuximab, and extralaryngeal spread (Table 2). Additionally, while the difference is not significant, patients with a low AUC60_p95_ tend to have worse DC (Fig. 2).

In the multivariable analysis with anatomical parameters (Table 3) only tumor volume ≥ 14.8 cm^3^ (HR=3.42, *P*<.001) remained significant for worse DC. In the multivariable model with DCE parameters no variables were significant.

**Table 3:** Multivariable analyses including anatomical MRI and clinical parameters for disease control and overall survival, n = 316.

**Patient and tumor characteristics**		Patients		**Disease Control**				**Overall Survival**		
				HR	95 % CI	*P-*value		HR	95 % CI	*P*-value
Age [years]	<65	146		NS				−		
	≥65	170						3.08	2.10–4.51	**<.001**
GTV volume [cm^3^]	<14.8	233		−				−		
	≥14.8	83		3.42	2.28–5.13	**<.001**		3.27	2.18–4.91	**<.001**
Tumor stage	T1	40		NS				−		
	T2	127						1.14	0.57–2.27	.71
	T3	117						1.48	0.73–2.99	.28
	T4a	27						2.27	0.97–5.27	.06
	T4b	5						9.21	2.83–29.93	**<.001**

Significant *P*-values are bold. Abbreviations: HR, hazard ratio; CI, confidence interval; NS, not significant.

### Overall survival

In the univariable analysis, the following factors were prognostic for a worse OS: age ≥ 65 years, tumor volume ≥ 14.8 cm^3^, tumor stage T4a and T4b compared to tumor stage T1, nodal disease, concurrent cetuximab, thyroid cartilage invasion, and extralaryngeal spread. Additionally, a AUC60_p95_ ≥ 31.7 mmol·s/L was prognostic for a better overall survival (Table 2).

In the multivariable analysis with anatomical paramters (Table 3), the following risk factors remained significant for a worse OS: age ≥ 65 years (HR=3.08, *P*<.001), tumor volume ≥ 14.8 cm^3^ (HR=3.27, *P*<.001), and tumor stage T4b compared to tumor stage T1 (HR=9.21, *P*<.001).

In the multivariable analysis with DCE parameters, a AUC60_p95_ ≥ 31.7 mmol·s/L remained significantly prognostic for a better OS (HR=0.25, *P*<.001, Table 4) when corrected for age, tumor volume, tumor and nodal stage, and treatment.

**Table 4:** Multivariable analysis including DCE-MRI and clinical parameters for overall survival, n = 89.

**Patient and tumor characteristics**		Patients		**Overall Survival**		
				HR	95 % CI	*P-*value
Age [years]	<65	36		−		
	≥65	53		4.48	1.92–10.45	**<.001**
Tumor stage	T1	11		−		
	T2	41		2.05	0.46–9.12	.35
	T3	29		6.17	1.35–28.22	.02
	T4a	8		25.74	4.68–141.66	**<.001**
AUC60_p95_ [mmol·s/L]	<31.7	18		−		
	≥31.7	71		0.25	0.12–0.53	**<.001**

Significant *P*-values are bold. Abbreviations: HR, hazard ratio; CI, confidence interval.

## Discussion

In this study, we examined the prognostic value of pretreatment radiological parameters. A low 95th percentile of the AUC60 in a tumor is the only MRI variable that was found to be significant in multivariable analysis and was prognostic for a worse OS. Other variables that were found to be prognostic in multivariable analysis were tumor volume for DC and OS, and age and T-stage for OS.

### DCE-MRI

A low AUC60_p95_ was prognostic for a worse OS, indicating that poorly perfused tumors lead to worse survival outcomes. Physiological imaging biomarkers like this could help guide treatment decisions. If more aggressive tumors can be identified pretreatment, concomitant therapy might be considered. Additionally, radiation dose might be increased to tumor areas that are likely to be more resistant to radiotherapy, e.g. areas with low AUC60_p95_. Another approach uses the change in DCE parameters during treatment to identify non-responsive tumor areas. A recent randomized controlled trial showed that a radiation boost to such tumor areas led to better local control [12]. Studies like this are promising for physiologically guided radiotherapy.

Only two other studies were found that studied the prognostic value of pretreatment area under the concentration curve in head and neck cancer on (chemo)radiotherapy treatment outcome [13], [14]. King et al. (2015) took the AUC of the entire scanning time (460 s), and found no correlation between the mean AUC and local control [13]. Chikui et al (2012) found no correlation between AUC of the scanning time (250 s) and treatment response in oral cancers [14]. Both of these studies did not include AUC_p95_ as a potential prognostic factors. In our study, the AUC60_median_ was also not prognostic for any of the outcome metrics evaluated, indicating that the peak perfusion might be more relevant to treatment outcome than the overall perfusion.

In this study, we performed a model-free analysis by calculating the AUC60. This is a robust and reproducible measure [15], [16], has a good signal-to-noise ratio, and does not require computationally demanding fitting of models. More importantly, it does not require an accurate read-out of the AIF, which is challenging on MRI [4]. However, while it does mirror biological mechanisms [4], [17], [18], the AUC does not have a direct physiological meaning [4], [19].

Model-based approaches can offer more insight into the underlying vasculature by modelling parameters like blood flow, intra- and extravascular space, and vessel permeability ($K^{\mathrm{trans}}$, transfer constant). However, the prognostic value of these parameters on radiotherapy treatment outcome in head and neck cancer remains unclear in literature. A recent systematic review identified three studies that evaluated the prognostic value of $K^{\mathrm{trans}}$ on OS [20]. While two studies found that a low $K^{\mathrm{trans}}$ was significant for worse OS [21], [22], one of them only included 20 patients [22]. The third study found no significant results [23]. For all other DCE factors and treatment outcomes, mixed results were found [20].

A limitation is the relatively small sample size of 89 patients that were included in the DCE analysis. The limited number of events in this group for LC and DC (15 and 28, respectively) might have been too small to find significant results. When analyzing the survival curves of Fig. 2, AUC60_p95_ has no effect on LC, but low AUC60_p95_ does seem to be associated with worse DC. This might indicate that tumors with low AUC60_p95_ have a higher chance of nodal and distant metastasis, contributing to the worse OS that was found. However in order to confirm this hypothesis, larger patient cohorts are necessary.

### Tumor volume

Tumor volume was a significant prognostic factor for DC and OS in multivariable analysis. We showed that tumor volume is an independent, significant prognostic factor when corrected for T-stage. This means that at every T-stage, larger tumors lead to worse treatment results. This was also found in our systematic review [1] and is in line with previous literature [24], [25], [26]. Tumor volume could therefore be a valuable biomarker when making treatment decisions. In clinical practice, tumor volume would mainly be of interest in the treatment of T3 tumors, where treatment guidelines tend to vary across centers [27]. Tumor volume should be considered when selecting patients with T3 tumors for concomitant chemotherapy.

### Anatomical tumor characteristics

Extralaryngeal spread was the only anatomical MRI characteristic that was significant for all outcome measures in univariable analysis. Thyroid cartilage invasion was significant for worse OS.

In our previous systematic review and *meta*-analysis, we found different results for the prognostic value of extralaryngeal spread on LC when determined on CT or MRI [1]. Extralaryngeal spread on CT was found to be a non-prognostic factor [28], [29], but when evaluated on MR it was deemed prognostic for worse LC [30], [31]. This is in line with the current analysis. It has been shown before that the diagnostic accuracy of extralaryngeal spread is limited on CT (ranging from 60-80 %) [32], [33], [34]. The reason for the discrepancy in prognostic value might thus be more accurate assessment of extralaryngeal spread on MRI [2].

In the previous review, we also found strong evidence for anterior commissure involvement being prognostic for worse LC [1]. This was not found in this current study. A possible explanation might be the inclusion of all tumor stages in our cohort, as anterior commissure involvement is more likely to be a relevant factor in smaller tumors. However, a subset of this cohort with early-staged tumors was previously analyzed, which also revealed no influence of anterior commissure involvement on LC [35].

In the multivariable analysis, no anatomical factors were found to be significantly prognostic for any of the included outcomes when corrected for tumor stage and other confounders. Most anatomical characteristics are directly or indirectly included in the determination of the tumor stage [36], so it is not surprising that they are not independent prognostic factors.

What is surprising is that we found no variables that were significant for LC in multivariable analysis. We assume this is because there were not enough local recurrences to show statistical significance.

The anatomical tumor characteristics in our cohort were assessed on either 1.5 T or 3 T MRI scans. While we believe both field strengths offer sufficient resolution to assess the anatomy, the use of imaging from different scanners might have influenced the results of our analysis.

## Conclusions

A low pretreatment AUC60_p95_ is prognostic for a worse OS in multivariable analysis and large tumor volume is prognostic for worse DC and OS. Both variables might be used to help guide treatment decisions in the future, but more data is needed to find adequate cut-offs.

Extralaryngeal spread is prognostic for worse LC, DC, and OS in univariable analysis. Additionally, thyroid cartilage invasion is prognostic for worse OS. In multivariable analysis, anatomical MRI parameters are not prognostic for any of the evaluated treatment outcomes when corrected for confounders like age, T-stage, N-stage, and tumor volume.

## Funding statement

This research was funded by the Dutch Cancer Society (KWF), project number 10978.

## CRediT authorship contribution statement

**Hilde J.G. Smits:** Methodology, Software, Formal analysis, Investigation, Data curation, Writing – original draft, Writing – review & editing, Visualization. **Saskia J. Vink:** Software, Investigation, Writing – review & editing. **Mischa de Ridder:** Methodology, Writing – review & editing, Supervision. **Marielle E.P. Philippens:** Conceptualization, Methodology, Software, Writing – review & editing, Supervision, Project administration, Funding acquisition. **Jan W. Dankbaar:** Conceptualization, Methodology, Investigation, Data curation, Writing – review & editing, Supervision.

## Declaration of competing interest

The authors declare that they have no known competing financial interests or personal relationships that could have appeared to influence the work reported in this paper.
