# Supplementary material for: Prognostic value of pretreatment radiological MRI variables and dynamic contrast-enhanced MRI on radiotherapy treatment outcome in laryngeal and hypopharyngeal tumors
Source: Clin Transl Radiat Oncol. 2024 Sep 12;49:100857. doi: 10.1016/j.ctro.2024.100857 (PMC11420635; doi:10.1016/j.ctro.2024.100857)
Supplement: Supplementary Data 1 [file mmc1.docx]

Supplementary Material 1 – Image acquisition parameters

**Supplementary Table S1:** MRI scanner and sequence parameters

| **Parameters** |  | |  |
| --- | --- | --- | --- |
| Median time interval between imaging and start treatment [days] | 14 days (range: 0-31) | |  |
| Scanner model | Intera 1.5 T or Ingenia 3.0 T | |  |
| Manufacturer | Philips Healthcare | |  |
|  |  |  |  |
| **MRI Parameters** | **T1w (n=325)** | **T2w (n=325)** | **DCE (n=90)** |
| Repetition time [ms] | 500-743 | 2107-7090 | 4.1-4.2 |
| Echo time [ms] | 14-20 | 100-130 | 2 |
| Slice thickness [mm] | 2-4 | 2-4 | 4 |
| Number of averages | 2 | 2 | 1 |
| Acquisition matrix | 252x180-  512x307 | 160x158-  512x299 | 168x168 |
| Acquired FOV [mm^2^] | 160x160-280x200 | 160x160-277.5x450 | 200x200 |
| Reconstruction matrix | 288x288-  512x512 | 288x288-  512x512 | 176x176 |
| Number of slices | 22-60 | 22-66 | 30 |
|  |  |  |  |
| Temporal resolution [s] | - | - | 4.50-5.01 |
| Number of dynamic scans | - | - | 19-60 |
| Contrast |  |  | 1 ml/s at 0.1 mmol/kg |
|  |  |  |  |

Supplementary Material 2 – Methods

*2.1 Image registration*

To correct for breathing and swallowing motions with the DCE-MRI time series, all individual scans were non-rigidly registered to a fixed DCE scan after contrast-enhancement. The registration was done using the Elastix toolbox^1,2^ and performed in the tumor area based on a volume of interest (VOI). This VOI consisted of the gross tumor volume delineation used in radiotherapy treatment that was expanded with an in-plane margin of 15 mm and a through-plan margin of 30 mm and cropped to stay within the body contour (Figure S1). The registration was optimized within the VOI.


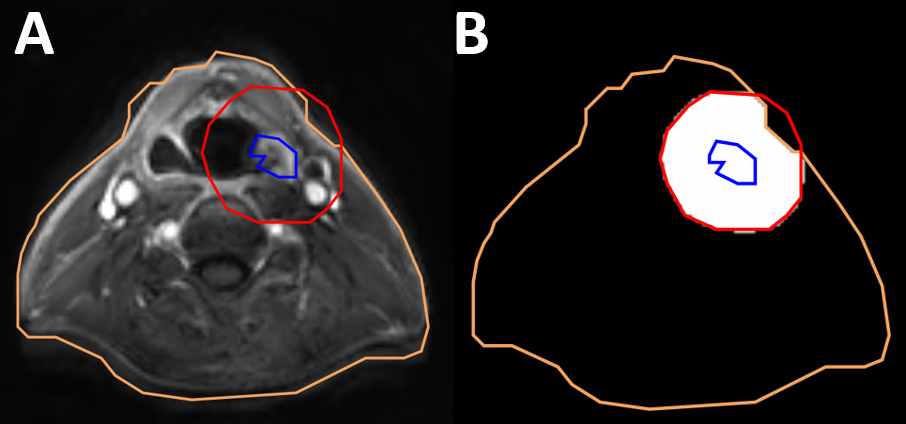

*Figure S1: Example of the volume of interest (VOI) for which the registration was optimized. The clinical delineation of the tumor (A, blue) was isotropically expanded with an in-plane margin of 15 mm (red). This expanded volume was cropped to the body contour (orange) to create the registration VOI of which a mask is shown in B.*

To check whether the registrations were successful, the structural similarity (SSIM) between the registered scans and the fixed scan was calculated before and after registration in Matlab R2019a (Mathworks, Natick, United States). The SSIM was only calculated within the VOI. If the SSIM became worse after registration (SSIM_registered_ – SSIM_original_ < -0.05), the original scan was used instead of the registered scan.

Poor registrations mainly happened in the early scans in the DCE time series, before the influx of contrast agent. Due to the lack of contrast agent, these scans contained less information and showed less similarities with the fixed scan that was contrast enhanced (Figure S2).


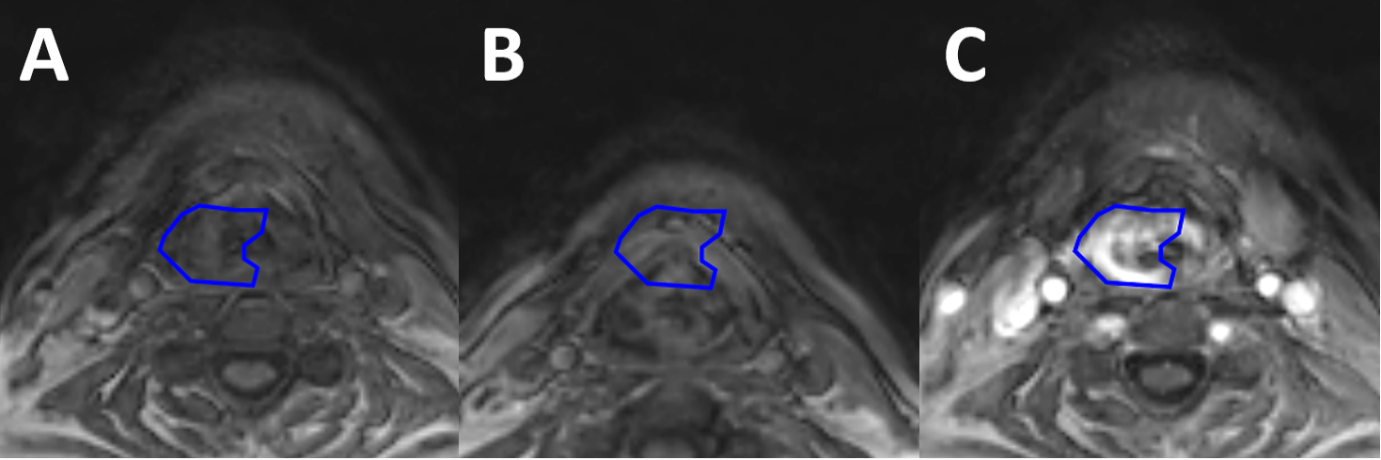

*Figure S2: Example of a poor registration. An early scan before influx of contrast (A) was registered to the fixed scan after the influx of contrast (C). B shows the resampled version of scan A based on the poor registration. The scan has shifted considerably backwards, causing the tumor contour (blue) to be in the wrong location. In these cases, the original scan A was used instead of the registered scan.*

*2.2 Start Area Under the Curve measurement*

To determine the start of the Area Under the Curve (AUC) measurement, we first determined the arterial input function (AIF) for each patient. The AIF was selected from the common or exterior carotid artery at the same side as the location of the tumor.

The peak of the AIF was determined and the start of the AUC measurement was chosen to be one time point before this peak. The temporal resolution of the scans was usually 4.95 seconds but ranged from 4.50 to 5.01 seconds. The start of the AUC measurement was thus chosen as ~5 seconds before the AIF peak (Figure S3).


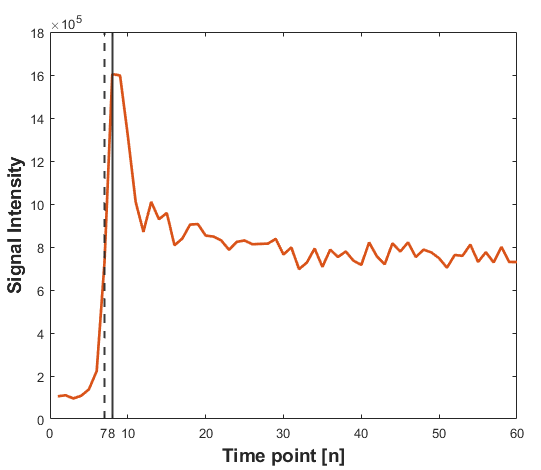


*Figure S3: Examples of arterial input function (AIF). The AIF peak of this patient was at time point 8 (solid horizontal line), so the AUC60 measurement was started at time point 7 (dashed horizontal line).*

*2.3 Data clean-up*

Laryngeal and hypopharyngeal tumors are likely to border air cavities or cartilage. Since it is impossible to calculate the AUC60 in these areas, it is likely that the AUC60 measurements in voxels around the edges of the tumor contain artifacts due to residual motion in the DCE scans.

To clean up the data, a histogram was made of the concentration values in all tumor voxels across all time points (Figure S4). The 2^nd^ and 98^th^ percentile (p2 and p98) values were calculated, which were -2.0 and 2.1 mmol/L, respectively. All voxels that had a contrast concentration <p2 or >p98 at any time point were removed from analysis, as the time concentration curve in this voxel was likely to contain an erroneous measurement.

Logically, 0 mmol/L would make more sense as a lower limit, as a negative contrast concentration is impossible. However, we found that many voxels contained negative concentration values before the influx of the contrast agent but showed a typical time concentration curve after contrast injection. Removing those voxels resulted in too much data loss, so we chose to use p2 as a lower limit.

After AUC60 calculation, an additional clean-up was performed where voxels with a negative AUC60 were removed from analysis.


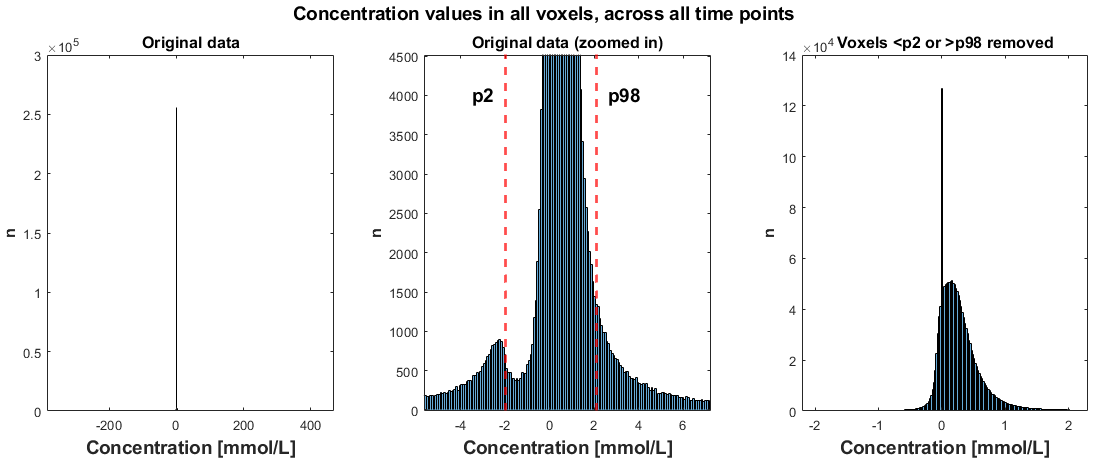


*Figure S4: Histograms of the contrast concentration in all tumor voxels of all patients across all time points (n≈1,700,000). The original data (left) shows a peak around 0. A cut-off of p2=-2.0 and p98=2.1 (middle) was used to clean up the data (right).*

After the clean-up of the data, 7.6% of tumor voxels were removed from analysis. All AUC60 maps were visually evaluated to check whether enough voxels remained for analysis.

In one patient, 97% of voxels were removed in data clean-up. The measured contrast concentrations in the DCE scans of this patient were significantly higher than in other patients. This patient was excluded from analysis.

In three other patients, 41%, 49% and 52% of voxels were removed. In these cases, the removed voxels were located in air cavities or cartilage (Figure S5). The voxels were thus justifiably removed. Since enough voxels remained that represented tumor area, the patients were still included.

In all other patients, less than 30% of voxels were removed in the data clean-up process.


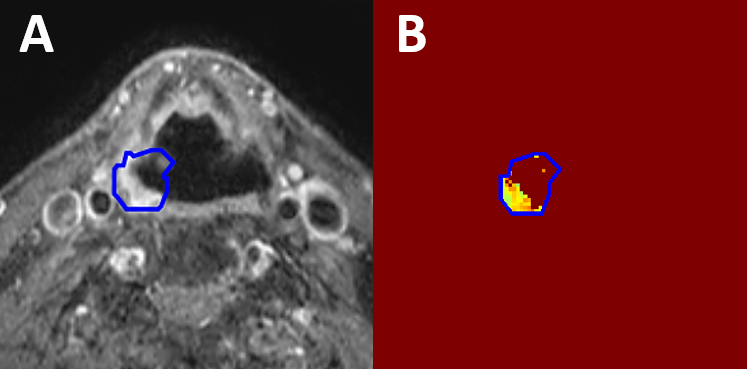


*Figure S5: Example of patient where a lot of voxels (52%) were removed in the clean-up of the data. The tumor delineation in blue includes a large air cavity (A). Since the AUC60 cannot be measured in this area, these voxels contained artifacts and were removed from analysis during data clean-up. In the AUC60 map (B, NaN-values in dark red) only 48% of voxels remain that actually represent tumor tissue.*

Supplementary Material 3 – Results

To check for multicollinearity in our model, the general variance inflation factor (GVIF) was determined for each variable. A GVIF-value above 5 is deemed to be unacceptable. We used the $GVIF^{\frac{1}{2*Df}}$ value, since our model contained categorical variables with more than one degree of freedom. For variables with only one degree of freedom, the $GVIF^{\frac{1}{2*Df}}$ equals $\sqrt{GVIF}$. Therefore, a $GVIF^{\frac{1}{2*Df}}$ value higher than $\sqrt{5}\approx2.24$ is deemed unacceptable. As shown in Supplementary Table S2, the variable paralaryngeal space involvement does not meet this criterion. This variable turns out to be strongly correlated to thyroid cartilage invasion (Pearson’s r = 0.89, Supplementary Table S3). To avoid unwanted effects, paralaryngeal space involvement was not included in the multivariable analysis. After this removal, the $GVIF^{\frac{1}{2*Df}}$ value of thyroid cartilage invasion dropped to 1.33.

***Supplementary Table S2:*** *Variance inflation factors*

|  | Generalized variance inflation factor (GVIF) | Degrees of freedom (Df) | $GVIF^{\frac{1}{2*Df}}$ |
| --- | --- | --- | --- |
| Age | 1.32 | 1 | 1.15 |
| Sex | 1.32 | 1 | 1.15 |
| Tumor location | 1.81 | 1 | 1.34 |
| GTV volume | 2.04 | 1 | 1.43 |
| Tumor stage | 6.04 | 3 | 1.35 |
| Nodal stage | 2.07 | 1 | 1.44 |
| Treatment | 2.03 | 2 | 1.19 |
| Thyroid cartilage invasion | 22.53 | 2 | 2.18 |
| Anterior commissure involvement | 1.44 | 1 | 1.20 |
| Pre-epiglottic space involvement | 1.33 | 1 | 1.15 |
| Paralaryngeal space involvement | 15.01 | 1 | 3.87 |
| Extralaryngeal spread | 2.66 | 1 | 1.63 |
| AUC60_median_ | 1.35 | 1 | 1.16 |
| AUC60_p95_ | 1.28 | 1 | 1.13 |

***Supplementary Table S3:*** *Cross-table thyroid cartilage invasion and paralaryngeal space involvement*

|  | | **Paralaryngeal space involvement** | |
| --- | --- | --- | --- |
|  |  | No | Yes |
| **Thyroid cartilage invasion scale** | Clear | 151 | 2 |
|  | Adjacent | 1 | 134 |
|  | Invasion | 0 | 32 |
